# On the reproducibility of hippocampal MEGA-sLASER GABA MRS at 7T using an optimized analysis pipeline

**DOI:** 10.1007/s10334-020-00879-9

**Published:** 2020-08-31

**Authors:** Yannik Völzke, Eberhard D. Pracht, Elke Hattingen, Desmond H. Y.Tse, Tony Stöcker

**Affiliations:** 1 German Center for Neurodegenerative Diseases (DZNE), Bonn, Germany; 2 Institute of Neuroradiology, University Hospital Frankfurt/Main, Frankfurt, Germany; 3 Neuroradiology, University Hospital Bonn, Bonn, Germany; 4 Department of Neuropsychology and Psychopharmacology, Faculty of Psychology and Neuroscience, Maastricht University, Maastricht, The Netherlands; 5 Department of Physics and Astronomy, University of Bonn, Bonn, Germany

**Keywords:** GABA, J-editing, 7Tesla, MEGA-sLASER, Reproducibility, Spectroscopy, Hippocampus

## Abstract

**Objectives:**

GABA is the most important inhibitory neurotransmitter. Thus, variation in its concentration is connected to a wide variety of diseases. However, the low concentration and the overlap of more prominent resonances hamper GABA quantification using MR spectroscopy. The hippocampus plays a pivotal role in neurodegeneration. Susceptibility discontinuities in the vicinity of the hippocampus cause strong *B*_0_ inhomogeneities, impeding GABA spectroscopy. The aim of this work is to improve the reproducibility of hippocampal GABA+ MRS.

**Methods:**

The GABA+/total creatine ratio in the hippocampus was measured using a MEGA-sLASER sequence at 7 Tesla. 10 young healthy volunteers participated in the study. A dedicated pre-processing approach was established. Spectral quantification was performed with Tarquin. The quantification parameters were carefully adjusted to ensure optimal quantification.

**Results:**

An inter-subject coefficient of variation of the GABA+/total creatine of below 15% was achieved. Additional to spectral registration, which is essential to obtain reproducible GABA measures, eddy current compensation and additional difference artifact suppression improved the reproducibility. The mean FWHM was 23.1 Hz (0.078 ppm).

**Conclusion:**

The increased spectral dispersion of ultra-high-field spectroscopy allows for reproducible spectral quantification, despite a very broad line width. The achieved reproducibility enables the routine use of hippocampal GABA spectroscopy at 7 Tesla.

**Electronic supplementary material:**

The online version of this article (10.1007/s10334-020-00879-9) contains supplementary material, which is available to authorized users.

## Introduction

GABA is the most important inhibitory neurotransmitter in the mammalian brain [1]. Changes of $\gamma$-aminobutyric acid (GABA) concentration are connected to a wide variety of diseases including schizophrenia [2], depression [3], and Parkinson’s disease [4]. However, GABA-MRS is hampered by its inherently low signal and the overlap of more prominent resonances. J-difference editing is often used to remove these overlapping resonances [5].

The GABA molecule contains three $\hbox {CH}_2$ groups which resonate at 1.9, 2.3 and 3.0 ppm. Between these nuclear spins, J-coupling is present. J-difference editing makes use of this coupling to separate the GABA signal and the overlapping resonances. This is done by subtracting two spectra where the GABA signal undergoes different J-coupling evolution. In one spectrum, the J-coupling evolution of the 3.0 ppm resonance gets refocused by selectively refocusing the 1.9 ppm resonance (edit-on); while in the other spectrum, the J-coupling evolution remains unperturbed (edit-off). The signal of overlapping resonances (e.g., creatine) remains unaffected and, thus, vanishes in the difference spectrum. However, the macromolecular signal is not completely suppressed. Thus, the measured signal, denoted by GABA+, is the sum of the GABA signal and a macromolecular component.

The hippocampus and its integrity is key to many cognitive and emotional functions. Alteration of its function or structure can be observed in various pathologies, including Alzheimer’s disease [6]. Furthermore, a large portion of astrocytes in the hippocampus contain GABA [7]. Animal models of Alzheimer’s disease show an increased release of GABA by reactive astrocytes [8, 9].

The hippocampus lies in proximity to air cavities within the sphenoid sinus and petrous bone. At these tissue boundaries, susceptibility discontinuities occur which cause strong $B_0$ field inhomogeneities. This results in a short $T_2^*$ that further hampers GABA quantification. To overcome this problem, the hippocampus is often only partially excited during a MRS experiment [10–12]. To our knowledge, no *J*-difference editing measurements were performed in the hippocampus so far.

Besides increased sensitivity, ultra-high-field spectroscopy also benefits from increased spectral resolution which allows a better metabolite separation. Especially, regions with severely distorted $B_0$ homogeneity benefit from the increased spectral resolution.

In this work, we investigate the reproducibility of GABA+ concentration measurements, resulting from MEGA-sLASER [13] experiments of the hippocampus. As a quality metric for the reproducibility, we investigate the inter-subject coefficient of variation of the GABA+/total creatine ratio in a group of young healthy volunteers. To improve the reproducibility intensive preprocessing, as well as fine-tuning of the spectral quantification process, is performed. During this optimization of the spectral quantification process, we minimize the intra-session coefficient of variation, i.e., the within-subject variation in repeated measurements (also known as repeatability).

## Methods

### Data acquisition

**Fig. 1:**
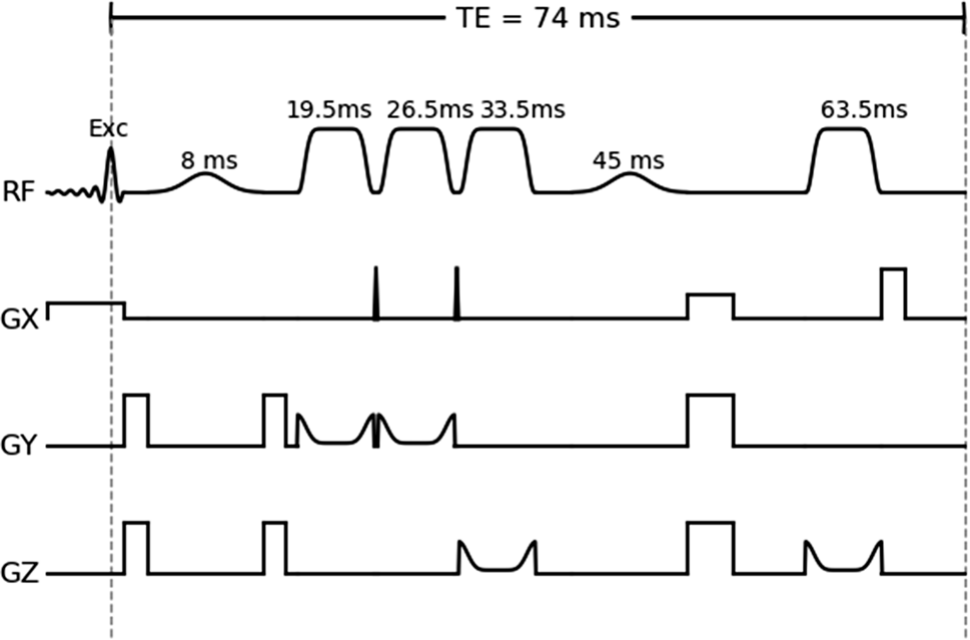
Optimized sequence diagram of the used MEGA-sLASER sequence. An asymmetric excitation pulse is followed by two pairs of slice-selective adiabatic refocusing pulses. Dual-band MEGA pulses are placed between the excitation and the first refocusing pulse, as well as in between the second pair of refocusing pulses. The pulse timing is shown above the respective pulses. Each repetition is preceded by a VAPOR water suppression module (not shown)

**Fig. 2:**
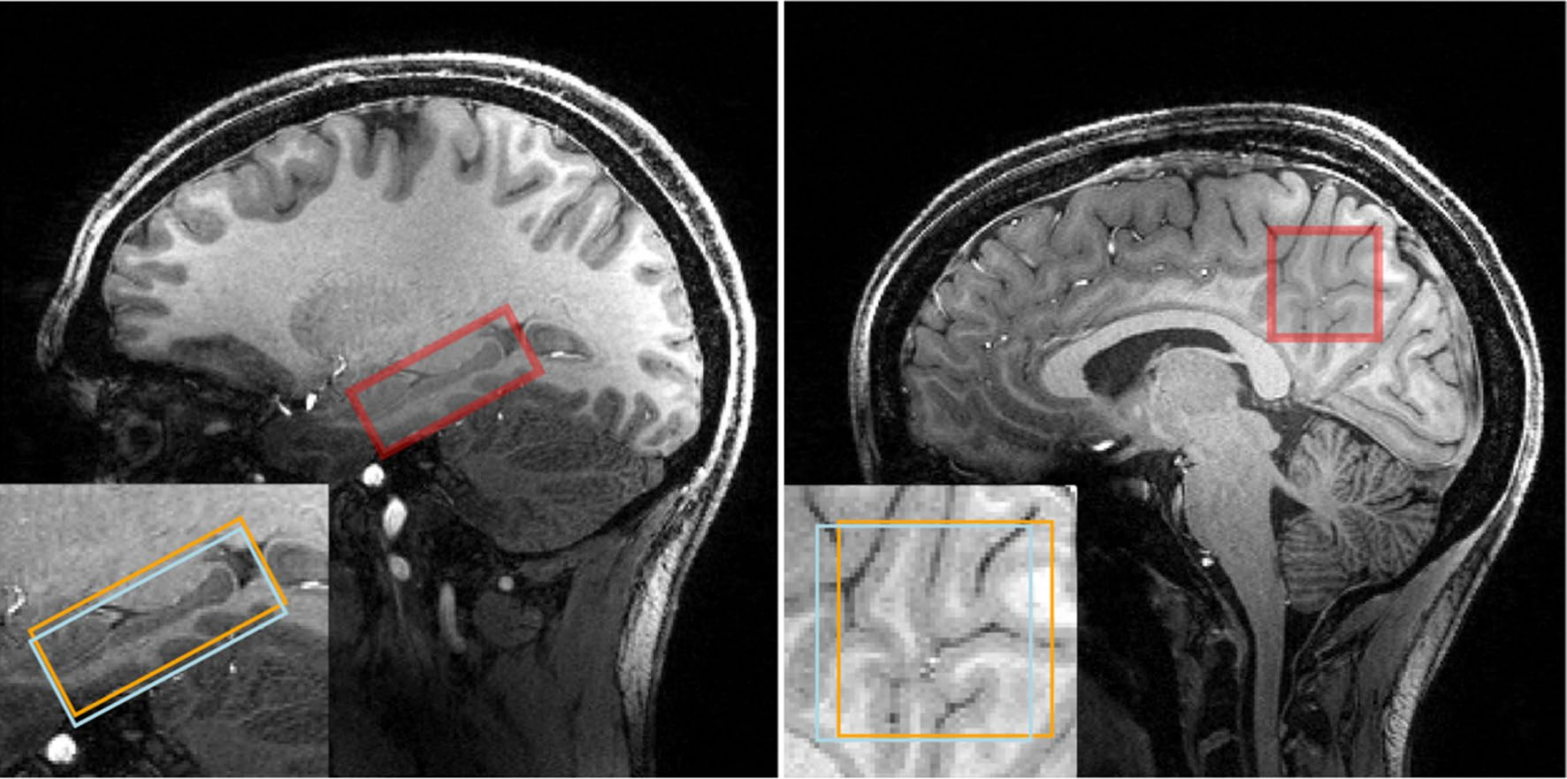
MP-RAGE images from one subject from the hippocampus data set (left) and one subject from the PCC data set (right). The nominal positions of the selected voxel are indicated by the red box. The (2 $\times$2 $\times$5) cm^3^ voxel is placed at the center of the hippocampus and aligned such that the long axis of the hippocampus is parallel to the voxel. A non-oblique (3$\times$3$\times$3) cm^3^ voxel is placed in the PCC. The insert shows the voxel positions of the GABA 3.0 ppm and the GABA 1.9 ppm resonance. The effective editing volume is the overlap of both

All measurements were performed on a 7T Magnetom (Siemens Healthineers, Erlangen, Germany) using a 32-channel head coil (Nova medical, Wilmington). The scanner is equipped with a gradient system allowing a nominal maximal gradient strength of 70 mT/m and a maximal slew rate of 200 T/m/s. GABA+ concentrations were estimated utilizing MEGA-semiLASER [13] acquisitions. Editing frequencies were set symmetrically around the water peak at 1.9 ppm (edit-on) and 7.5 ppm (edit-off). To optimize coherence pathway selection, crusher gradient moments were maximized within the hardware limits. Water suppression was performed using VAPOR [14], in combination with dual-band editing pulses [5]. The corresponding sequence diagram is depicted in Fig. 1.

10 young healthy volunteers ($26.6 \pm 4.7$ years, 5 females) participated in this study. Written informed consent was given by all subjects before examination. A whole-brain, high-resolution, T1-weighted MP-RAGE [15] image was acquired for each subject. This image was used to guide the placement of a (2 $\times$ 2 $\times$5) cm^3^ voxel centered around the hippocampus, as shown in Fig. 2. Afterwards, the $B_1$ amplitude was manually adjusted based on $B_1$ mapping, utilizing 3DREAM [16]. The sLASER localization is quite insensitive against $B_1$ variation. However, this is not the case for the MEGA pulses, rendering careful $B_1$ adjustments necessary. Three circular regions of interest, concentric to the spectroscopic voxel in different orientations, were defined roughly of the size of the voxel. Based on the $B_1$ map, the voltage needed for perfect refocusing within these regions was calculated and manually set. Subsequently, an automated 2-step $B_0$ shimming procedure was applied. A GRE-based $B_0$ map was acquired and shim values were calculated based on the selected spectroscopic voxel. Using the calculated shim currents, this procedure was repeated in a second step.

Finally, three consecutive spectroscopy measurements were performed, utilizing an in-house-developed MEGA-semiLASER sequence. The pulse center of the pulses is fixed to match the timing presented in [13], resulting in $T_\mathrm{E}=74\text { ms}$. After an asymmetric excitation pulse (3.3 kHz), the sequence performs adiabatic refocusing using GOIA-WURST pulses (6.7 ms, 16.8 kHz, 18 $\upmu \hbox {T}$) [17]. The pulses were detuned to 2.4 ppm, right between the outermost GABA resonances. The high bandwidth causes a chemical shift difference error (CSDE) of 2% (refocusing) and 10% (excitation) between these resonances. To optimize the inversion profile, the pulse duration was maximized. Thus, the pair of *z* axis spoiler gradient pulses could not be included due to the lack of available time. Besides this, the gradient scheme is exactly as presented in [13]. The last crusher gradient (33.3 mT/m, 260 $\upmu \hbox {s}$ ramptime, 6.25 ms flat top) is switched off directly before the acquisition, generating substantial eddy currents. Therefore, an additional eddy current compensation (on top of the vendor provided one) was implemented in the data processing workflow (see preprocessing section). The bandwidth of the 8.2-ms-long editing pulses was slightly higher than in [13] (190 Hz instead of 130 Hz). Due to SAR limitations, $T_\text {R}=7\,\hbox {s}$ was required. During the first excitation of each measurement, water suppression was omitted to obtain a water reference scan. The total acquisition time was 8:03 min (water reference, 4 dummy excitation, 32 on- and 32 off-acquisition).

To better assess the quality of the hippocampus spectra, the same acquisition protocol was used in a reference study. 3 young healthy volunteers ($28.3 \pm 2.1$ years, 2 females) participated in this study. A (3$\times$3$\times$3) cm^3^ voxel in the posterior cingulate cortex (PCC) was selected as presented in [18].

### Preprocessing

Preprocessing was performed using an in-house-developed toolbox. First, the raw data were split into water-suppressed and water-unsuppressed data. Both datasets were used for adaptively optimized combination [19] of the individual coil signals. The complex water signal amplitude, $s_i$, for each coil and the noise correlation matrix, ${\mathcal {N}}_{i,j}$, can be extracted from the water-suppressed and water-unsuppressed signal. The coil weights are defined as $w_i = ({\mathcal {N}}^{-1})_{i,j}s_j$.

The vendor-provided eddy current compensation was performed prior to the preprocessing routine. However, to minimize residual eddy current effects, an additional, subsequent eddy current compensation (ECC) [20] was applied, using the coil-combined water-unsuppressed FID. Afterwards, spectral registration (SR) [21] was applied to remove phase and frequency variations of the individual excitations. The signal of the *j*-th excitation $A_j(t)$ was modified before averaging, according to

1 $$\begin{aligned} \begin{aligned} A_j^\text {SR}(t,\phi _j,f_j)&= A_j(t) \exp {\left( i\phi _j+2\pi if_jt\right) }\,\,\text {with}\\ (\phi _j,f_j)&= \underset{\phi ,f}{\text {argmin}}\Vert A_j^\text {SR}(t,\phi ,f) -R(t) \Vert _2, \end{aligned} \end{aligned}$$

where *R*(*t*) is the reference signal. The mean of all edit-off signals was used as reference. Spectral registration can also be used for difference artifact suppression by phase and frequency correcting the averaged edit-on signal with respect to the averaged edit-off signal before subtraction. This will be abbreviated by DAS in the following sections. The frequency span (2.8, 3.5) ppm was selected as described in [21]. The phase and frequency correction can be calculated as

2 $$\begin{aligned} (\phi _\text {on},f_\text {on}) = \underset{\phi ,f}{\text {argmin}}\Vert A_\text {on}^\text {SR}(t',\phi ,f) -A_\text {off}^\text {SR}(t') \Vert _2. \end{aligned}$$

For comparison, the similar, recently proposed difference optimization (DO) [22] method was also implemented. A flowchart of the presented preprocessing approach can be found in Supplementary Figure S1. Consequently, six different preprocessing approaches were tested:

1. only spectral registration (SR)
2. eddy current compensation and spectral registration (ECC+SR)
3. spectral registration and difference artifact suppression (SR+DAS)
4. eddy current compensation, spectral registration and difference artifact suppression (ECC+SR+DAS)
5. spectral registration and difference optimization (SR+DO)
6. eddy current compensation, spectral registration and difference optimization (ECC+SR+DO)

Finally, the edit-off and the difference signals were exported in the JMRUI file format [23] for spectral quantification.

### Spectral quantification

Spectral quantification was performed using TARQUIN [24]. To calculate the GABA+/total creatine ratio, the edit-off and the difference signal had to be quantified individually. TARQUIN’s internal sLASER basis set was used to quantify the edit-off signal. Here, the basis set is calculated from the simulated time evolution of the density matrix during an idealized sLASER sequence with matching TE. The internal MEGA-PRESS basis set contains singlet signals only and the GABA pseudo-doublet is described by two individually treated singlets. This approximation is valid not only for MEGA-PRESS data, but also for MEGA-sLASER data. However, we implemented a modified version of TARQUIN’s internal MEGA-PRESS basis set to quantify the difference signal. To better capture co-edited signals, additional resonances were included at 2.5 and 2.7 ppm.

TARQUIN uses multiple shaping parameters. It was found that the choice of the initial guess for the strength of the Gaussian decay, $\beta _\mathrm{s}$, (*init_beta* in TARQUIN) and the first FID point that is used for quantification, $n_\mathrm{s}$, (*start_pnt* in TARQUIN) affect the quantification significantly.

After optimization of the fitting parameters, data quality was assessed by calculating the FWHM of the dominant resonance (NAA). Furthermore, the SNR of the NAA resonance was calculated, defined as: The peak signal of the baseline-corrected spectral fit divided by two times the root mean square of the spectrum in a region without resonances.

### TARQUIN parameter optimization

The optimization was performed as a two-step process. First, $\beta _\mathrm{s}$ and afterwards, $n_\mathrm{s}$ was optimized. In total, 13 different $n_\mathrm{s}$ values between 1 and 50, and 10 different $\beta _s$ values between 200 and 5000 were tested. During both optimization steps, the intra-session coefficient of variation, $\text {CoV}^\text {intra}$, was calculated for each subject. As a quality metric for the quantification stability, we utilized the mean intra-session coefficient of variation, $\text {mCoV}^\text {intra}$, over all subjects.

For the optimization process of $\beta _\mathrm{s}$, two different preprocessing routines were used. Standard processing (SR) was similar to the processing applied in a recent multi-site study [18]. For advanced processing, ECC + SR + DAS was used. For both processing approaches, $\text {mCoV}^\text {intra}$ was calculated for each 13$\times$13 = 169 pairs of $n_\mathrm{s}$. The median values of these $\text {mCoV}^\text {intra}$ were calculated for every $\beta _\mathrm{s}$ and both processing routines. The value of $\beta _\mathrm{s}$ that minimizes the average of these values was selected. Afterwards, the $\text {mCoV}^\text {intra}$ was calculated for all 169 pairs of $n_\mathrm{s}$ values and all six preprocessing approaches. The pair of $n_\mathrm{s}$ values that minimizes the median $\text {mCoV}^\text {intra}$ over the preprocessing approaches was selected.

## Results

### Data quality

**Fig. 3:**
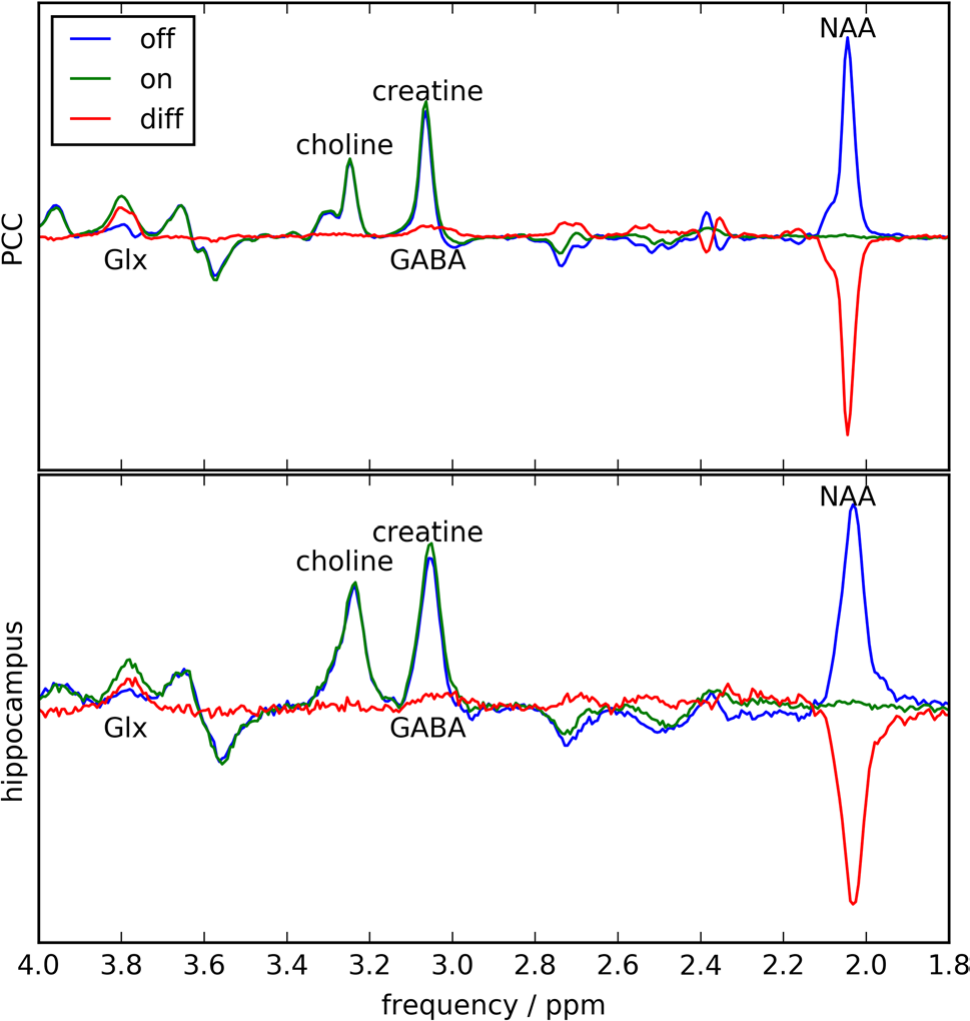
Edited, unedited and difference spectra of one subject in the PCC study (top) and on subject in the hippocampus study (bottom). Besides a much broader line width and decreased SNR in case of the hippocampus data, the results of both brain regions look very comparable

**Fig. 4:**
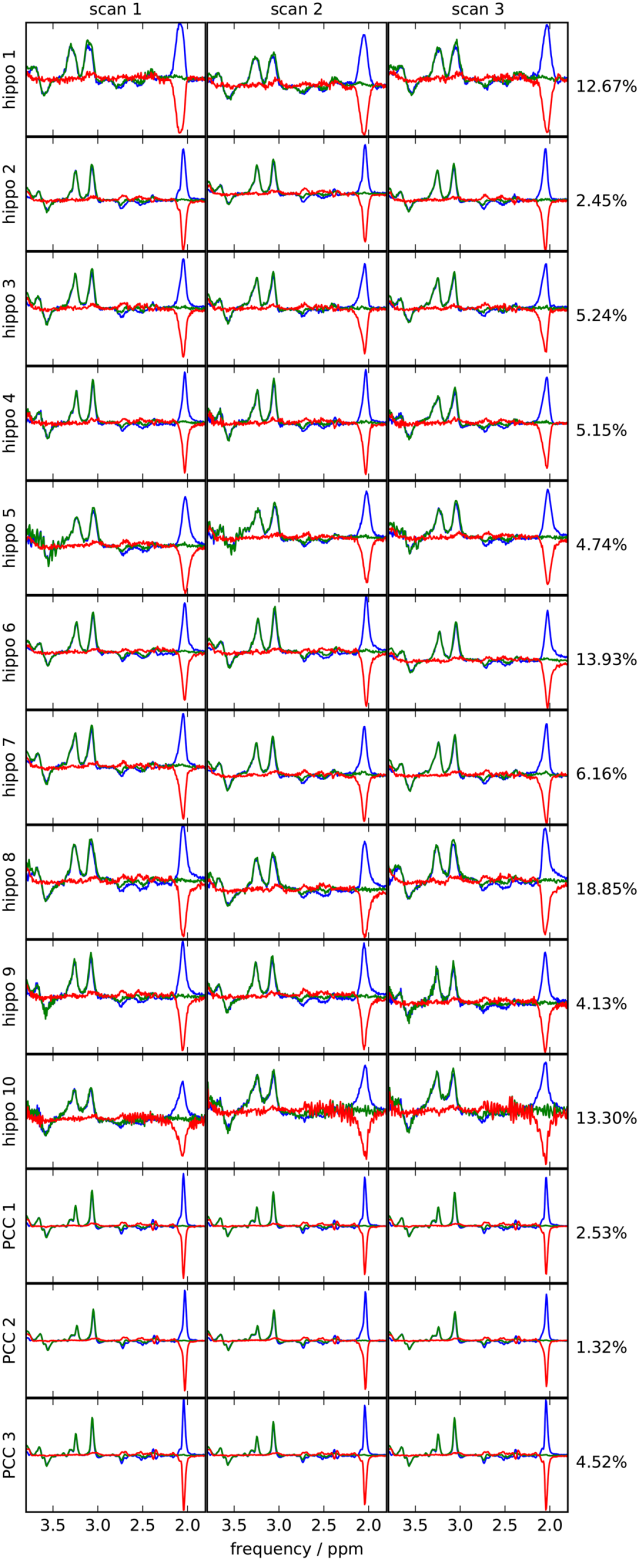
Edited, unedited and difference spectra of all measurements of hippocampus study (10 subjects) and the PCC study (3 subjects). Right to the plots, the intra-session CoV of the GABA+/ total creatine ratio in the respective subject

Visual inspection of the data quality was carried out on every spectrum. In nine out of ten subjects, no artifacts were found. However, spurious echoes were observed in one subject. Data from this subject were reacquired. One representative acquisition from each brain region is depicted in Fig. 3. A complete overview of all measured spectra is provided in Fig. 4.

The mean NAA line width within the hippocampus data set was 22.26 Hz for the diff-spectrum, and 22.74 Hz for the off-spectrum. Within PCC the mean line width was 8.68 Hz (diff-spectrum), and 8.88 Hz (off-spectrum). The mean NAA-SNR of the hippocampus data was 35.1 (diff-spectrum) and 43.3 (off-spectrum); while for the PCC, the mean SNR was 168.0 (diff) and 204.0 (off).

Accounting for the volume difference between the hippocampus voxel (20 ml) and the PCC voxel (27ml), as well as the broader line width within the hippocampus, a SNR disparity of a factor of approximately 1.35 remains.

### TARQUIN parameter optimization

In the first step, $\beta _\mathrm{s}$ was optimized. The intra-session reproducibility was strongly affected by this value and a maximum at $\beta _\mathrm{s} = 1500$ is reached. The effect on the reproducibility of $n_\mathrm{s}$ is smaller. However, using this $\beta _\mathrm{s}$, the intra-session reproducibility was maximized by $n_\mathrm{s}$ = 3 for the difference- and $n_\mathrm{s}$=4 for the off-signal quantification. As expected, because of the longer $T_2^*$, smaller optimal $\beta _\mathrm{s}$ and larger optimal $n_s$ were calculated for the PCC. These results are summarized in the Supplementary Figure S2.

**Fig. 5:**
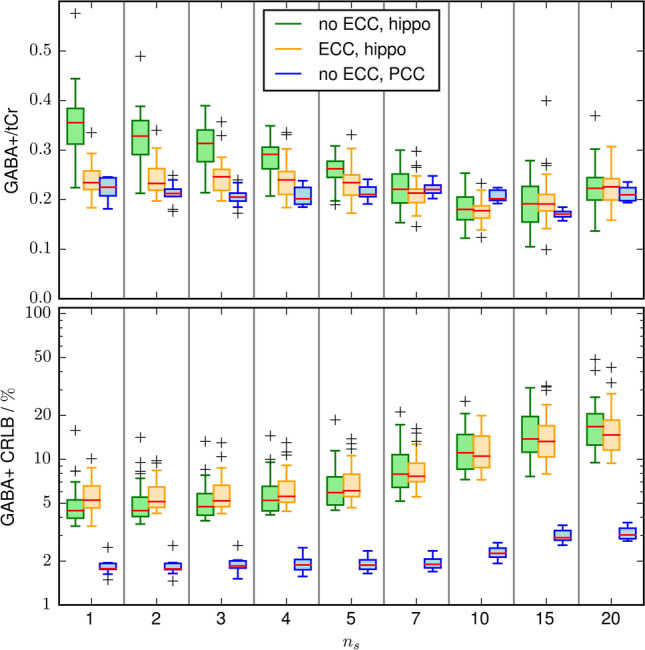
Top: Boxplots of the GABA+/total creatine ratio using spectral registration and difference artifact suppression as a function of the FID start points. The results of the hippocampal dataset with and without eddy current compensation, are depicted in green and orange, respectively. The quantification results are biased by the chosen $n_\mathrm{s}$. This bias is significantly reduced by applying ECC. As a comparison the GABA+/total creatine ratio is also shown for the PCC dataset (blue). The quantification results are almost independent of $n_s$. Bottom: the CLRB of the GABA+ quantification. Omitting the first data points results in loss of SNR, which cause an increase of CRLB. Due to the much higher SNR the CRLB are much smaller for the ECC data set

Despite the small effect on the reproducibility, $n_\mathrm{s}$ significantly affects quantification, as depicted in Fig. 5. In the top plot, for each $n_\mathrm{s}$, the GABA+ / total creatine ratio is calculated and all 30 measurements in the hippocampus and 9 measurements in the PCC are summarized in boxplots. In the bottom plot, the Cramer–Rao lower bounds (CRLB) of the GABA+ quantification are depicted in the same fashion.

Without ECC, strong variations of the measured hippocampal GABA+ / total creatine are visible for small $n_\mathrm{s}$. Applying ECC significantly reduces these variations. For $n_\mathrm{s} > 5$, comparable quantification results were achieved with and without ECC. The measured GABA+/ total creatine ratio in the PCC is much less affected by $n_\mathrm{s}$.

The first points in the FID have the highest signal. Thus, omitting them causes an SNR reduction which leads to increased CRLB. This is depicted in the bottom plot of Fig. 5. As there are more high SNR points in the PCC FIDs, due to the longer $T_2^*$, the CRLB increase is much slower.

### Preprocessing

**Fig. 6:**
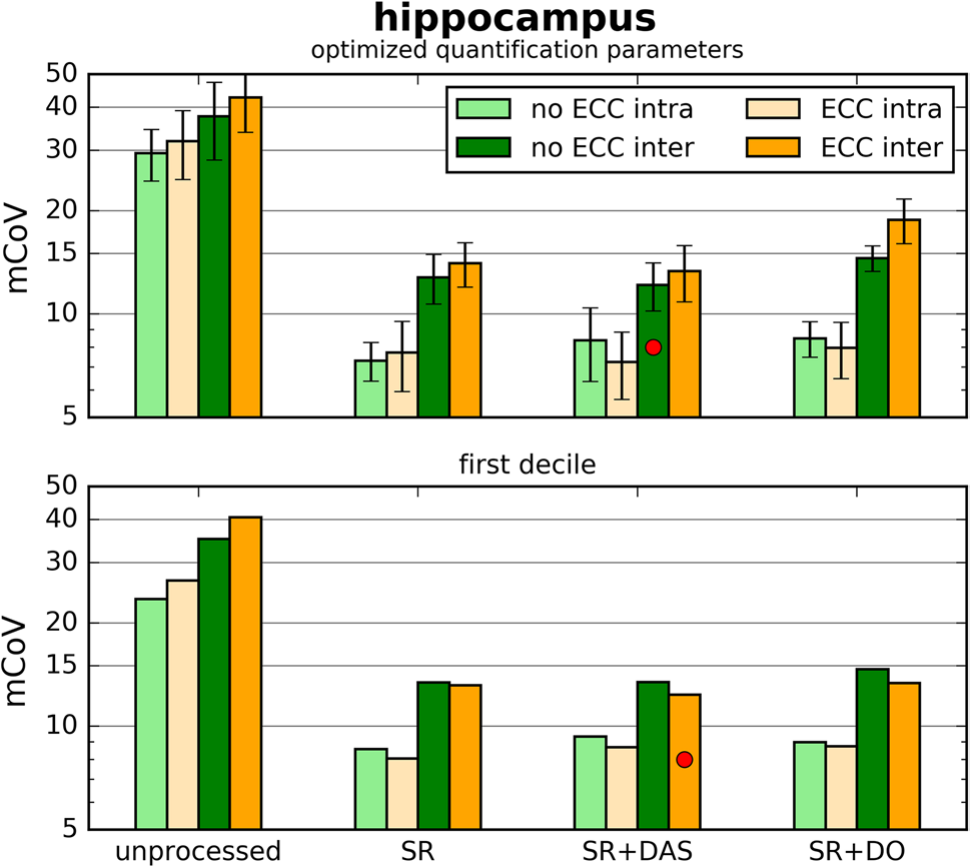
Mean hippocampal inter-subject (dark colors) and intra-session (light colors) coefficient (mCoV) of variation obtained with various preprocessing routines. Top: mCoV, calculated with optimized quantification parameters; Bottom: first decile of mCoV for each preprocessing routine. Both metrics show similar results. Spectral registration reduces the mCoV significantly. The quantile method indicates ECC + SR + DAS as the optimal processing routine; using the optimized quantification parameters indicates SR+DAS as optimal

Figure 6 shows the mean intra-session coefficient of variation $\text {mCoV}^\text {intra}$ and the mean inter-subject coefficient of variation $\text {mCoV}^\text {inter}$ for the different preprocessing approaches. The top plot depicts the coefficient of variations obtained with the optimized quantification parameters. Applying simple averaging, without dedicated preprocessing, leads to mCoVs above 30%. The $\text {mCoV}^\text {intra}$ of all six preprocessing approaches is below 10% without significant variation.

Application of SR reduces the $\text {mCoV}^\text {inter}$ from around 40% to below 15%. The $\text {mCoV}^\text {inter}$ for each preprocessing approach match within the errors. The minimal $\text {mCoV}^\text {inter}$ was achieved using SR+DAS (12.1%).

For each preprocessing approach, 169 pairs of $n_s$ values were tested. For each pair the coefficient of variation was calculated. The bottom plot of Fig. 6 shows the top decile of these values. Again, the $\text {mCoV}^\text {intra}$ of all preprocessing approaches is below 10%. The variation between the approaches is decreased compared to the top plot. Applying ECC reduces the $\text {mCoV}^\text {inter}$ of every preprocessing approach. The approach ECC+SR+DAS leads to the smallest $\text {mCoV}^\text {inter}$ (12.3%).

## Discussion

This study demonstrates that hippocampal GABA+ concentration can be measured with a high reproducibility using MEGA-sLASER at 7 Tesla. Optimization of the preprocessing routine and the quantification process minimizes the inter-subject CoV to below 15%.

The increased spectral dispersion of ultra-high field allows a better metabolite separation. In this work, the increased spectral dispersion enables spectral quantification of signal from a voxel with severe $B_0$ inhomogeneities. The FWHM is a commonly used quality criterion and spectra with a FWHM of more than 0.1 ppm are often suggested to be disregarded [25, 26]. The maximal NAA-FWHM in this work was 29.3 Hz (0.099 ppm) and the mean FWHM was 22.7 Hz (0.076 ppm).

As the NAA line width is extracted from the spectral fit, the mean NAA line extracted from the off and diff signal differs slightly. The SNR extracted from the two fits, however, differs strongly as the difference signal originates from the subtraction of two noisy signals. The SNR of the hippocampus data is lower by a factor of 4.7–4.8, compared to the PCC data. This can be explained to a large part by the broader line widths and the smaller voxel size. With only these two effects a factor of 3.5 would be expected. Presumingly, the remaining SNR loss can be attributed to the coil sensitivity. The hippocampus lies farther within the head than the PCC, resulting in a larger distance to the dominant receive coils.

Overlapping resonances from other metabolites vanish in the subtraction, during a J-difference editing experiment. However, this is not the case for some macromolecular resonances. Suppression of these macromolecular signal requires additional acquisition techniques. Two commonly used methods are either based on inversion recovery [27] or setting the resonance frequencies of the MEGA pulses symmetrically around the macromolecular resonance of 1.7 ppm [28]. Inversion recovery leads to a substantial loss of SNR. Symmetrical placing of the editing frequency reduces the macromolecular contribution without affecting the GABA signal. However, it was shown that this method significantly lowers the reproducibility at 3 Tesla [18]. As the reproducibility is already reduced in the hippocampus by the short $T_2^*$, we omitted macromolecular suppression in this work. On the the other hand, this method could strongly benefit from the increased spectral dispersion at 7 Tesla. Therefore, macromolecular suppression would be a very interesting addition for future studies.

7-Tesla spectroscopy benefits from increased signal strength. For most metabolites, the shorter $T_2$ times reduces the signal strength when long echo times are used. However, the reported the GABA $T_2$ at 7 Tesla are 87 ms [29] and 63 ms [30] which are very similar to the reported 88 ms at 3 Tesla [31]. Thus, no substantial loss of SNR is to be expected.

It was found that the MEGA-sLASER is prone to spurious echoes. To tackle this problem, crusher gradient moments were maximized within the hardware limits. However, this method is not failsafe as spurious echoes occur in one subject. If eddy currents remain after the vendor-provided ECC, a quantification bias is introduced. By applying additional, subsequent ECC this effect can be removed. However, this bias is only visible in the hippocampus data. Presumingly, this is because of the shorter $T_2^*$. Only the very first FID points are corrupted by the eddy currents. Therefore, especially the quantification of the hippocampus data might be more prone to eddy currents as there are less high SNR points. For the same reason, the quantification bias vanishes for greater $n_\mathrm{s}$.

Phase and frequency variations between the excitations cause residual creatine signal in the difference spectrum. This difference artifact is mistaken as GABA+ signal during spectral quantification. Spectral registration removes most of these variations. This is the most important preprocessing step for reproducible GABA+ quantification. Although there is only a small increase of precision when adding eddy current suppression, it is essential to obtain accurate GABA+ measures. Difference artifact suppression performs an additional phase and frequency modulation prior to signal subtraction. Only subtle changes in the reproducibility were achieved due to this preprocessing routine. However, difference artifact suppression did increase the reproducibility in both quality metrics for both enabled and disabled ECC. In conclusion, the optimal preprocessing routine for our data is ECC+SR+DAS.

TARQUIN assumes Voigt shape resonances, where the Gaussian decay accounts for dephasing due to intra-voxel $B_0$ inhomogeneities. The starting value of decay strength $\beta _\mathrm{s}$ significantly affects the reproducibility of the spectral quantification. If $\beta _\mathrm{s}$ is far from the optimum, the spectral quantification is prone to local minima. Thus, a careful adjustment is needed. In contrast, the reproducibility of the spectral quantification is only slightly influenced by the start point of quantification, $n_\mathrm{s}$, despite higher CRLB. Thus, we conclude that the systematic changes of the measured GABA+/total creatine ratio for different $n_\mathrm{s}$ originate from eddy currents and not from fit instabilities.

Multiple reproducibility studies were performed at lower field strength in less challenging brain regions. Commonly, the inter-subject coefficient of variation of the GABA+/total creatine ratio is used as quality metric which is usually around 10% [18, 32]. Suppression of macromolecular signal increases the coefficient of variation to 13–20% [18, 33]. The test–retest coefficient of variation can be smaller and strongly depends on the time between scans [34]. The inter-subject coefficient of variation includes methodological inaccuracies, as well as biological variations. These two effects cannot be easily separated. The test–retest method reduces the biological variations by comparing measurements of the same subject. The same holds true for the intra-session reproducibility. However, it additionally reduces possible methodological inaccuracies as identical $B_0$ shim, $B_1$ calibration and voxel placement are used for the repeated measurements. Therefore, the intra-session CoV forms an upper bound, while the inter-subject CoV is the lower bound of the expected test–retest reproducibility.

Using J-difference editing at 7T Wijtenburg et al. reported test–retest CoVs of 16.2% and 13.4% in the anterior cingulate and the dorsolateral prefrontal cortex, respectively [35]. Prinsen et al. reported a test–retest CoV of 9.5% in the occipital cortex [36]. Prinsen et al. utilized a MEGA-sLASER sequence, while Wijtenburg et al. used MEGA-PRESS with inner volume suppression. The usage of MEGA-sLASER leads to an improved inversion profile which might explain the improved reproducibility. Additionally, Prinsen et al. used LC model for spectral quantification while Wijtenburg et al. did peak integration. This might also be a reason for the improved reproducibility. The averaged line width reported by Prinsen et al. was 12.2 Hz, while Wijtenburg et al. could achieve a line width below 10 Hz in both examined regions. Thus, the reproducibility of the hippocampal GABA+ measurements in this work is comparable to the reported values from other brain regions, despite of a much broader line width. However, both studies used macromolecule suppression, which was found to lower the reproducibility at 3 Tesla [18].

Due to the increased spectral resolution of ultra-high-field spectroscopy, GABA concentration can also be extracted from short-TE sequences reliably at 7 Tesla. Utilizing short-TE STEAM, Wijtenburg et al. achieved a lower test–retest CoV in the anterior cingulate but a higher CoV in the dorsolateral prefrontal cortex, compared to J-difference editing. Prinsen et al. achieved comparable reproducibility with MEGA-sLASER and short-TE STEAM. However, the broad line width of hippocampus spectra limits the spectral resolution. Consequently, the spectral resolution of the hippocampus spectra is comparable to 3-Tesla studies in less challenging brain regions. Therefore, short-TE spectroscopy is not a promising approach to measure hippocampal GABA concentrations at 7 Tesla.

The big difference in reproducibility for the investigated brain regions suggests that the main error source is the limited data quality of the hippocampus spectra. Data quality is corrupted by the difficult shim conditions for the hippocampus which results in a short $T_2^*$. Improvements of the shimming results could possibly be achieved by the use of higher order shim coils [37].

An intrinsic downside of adiabatic sequences at 7 Tesla is the high SAR. $\mathrm{TR} = 7$ s was required to comply with the SAR limitations enforced by patient safety regulation. A lower repetition time would lead to a more efficient sampling and, thus, an increased SNR. Probably SAR minimization by $B_1$ shimming [38] is the most promising approach reducing TR.

Combining $B_1$ shimming and improved $B_0$ shimming could lead to strongly increased data quality and, thus, significantly higher reproducibility of hippocampal GABA+ quantification.

Partial volume effects are an additional limitation of the presented study. Only approximately 20% of the volume of interest is actual hippocampus tissue (calculated via hippocampus segmentation of the MPRAGE using FSL [39]). Signal from tissue surrounding the hippocampus contribute to the measured GABA+/total creatine ratio. This includes other parts of the allocortex, like the parahippocampal gyrus, which are functionally closely linked to the hippocampus [40]. Partial volume effects will further increase in the presence of hippocampus atrophy, which is a common symptom in neurological diseases. The dimensions of the voxel are given by the shape of the hippocampus and the limitation of cuboid voxel selection. To reduce partial volume effects, and thus increase the sensitivity on changes in hippocampal GABA concentration, subject-specific localization techniques need to be explored. Here, parallel-transmit-based [41] approaches may become feasible in the near future. Nevertheless, the sensitivity of the presented approach may be sufficient for the investigation of disease-specific changes of GABA+ concentrations. This will be explored in upcoming clinical cohort studies on patients with neurodegenerative diseases.

## Conclusion

It was shown that MEGA-sLASER at 7 Tesla enables reproducible measurements of the GABA+/total creatine ratio with an inter-subject CoV of around 12%. This is comparable to the reported values from less challenging brain regions. To achieve this level of reproducibility, dedicated preprocessing had to be established. Furthermore, the quantification process had to be carefully adjusted. The achieved reproducibility allows for routine use of whole-hippocampus GABA+ spectroscopy in clinical studies.

## Electronic supplementary material

Below is the link to the electronic supplementary material.

Supplementary figure 1: Flowchart depicting data preprocessing. Raw data are read in and split into water-suppressed and water-unsuppressed signal. Both signals are used for coil combination. In the red box, optional preprocessing steps are shown. This includes eddy current compensation, spectral registration and difference artifact suppression. The resulting processed data are exported in JMRUI file format and imported by TARQUIN for spectral registration

Supplementary figure 2: Results of TARQUIN parameter optimization. Left: median intra-session mCoV over all pairs of $n_s$ as a function of $\beta _s$. It was calculated for the processing routines SR (standard processing) and ECC + SR + DAS (advanced processing). The minimum of the average of this two values were reached at $\beta _s=1500$ (hippocampus) and $\beta _s=600$ (PCC). Right: median intra-session mCoV over all processing approaches for various combinations of $n_s$, using optimized $\beta _s$. The white circles indicate the minima for spectral quantification of the difference- and off-signal quantification of the respective brain region
