# Supplementary figures and images for: On the reproducibility of hippocampal MEGA-sLASER GABA MRS at 7T using an optimized analysis pipeline
Source: MAGMA. 2020 Aug 31;34(3):427–36. doi: 10.1007/s10334-020-00879-9 (PMC8154804; doi:10.1007/s10334-020-00879-9)

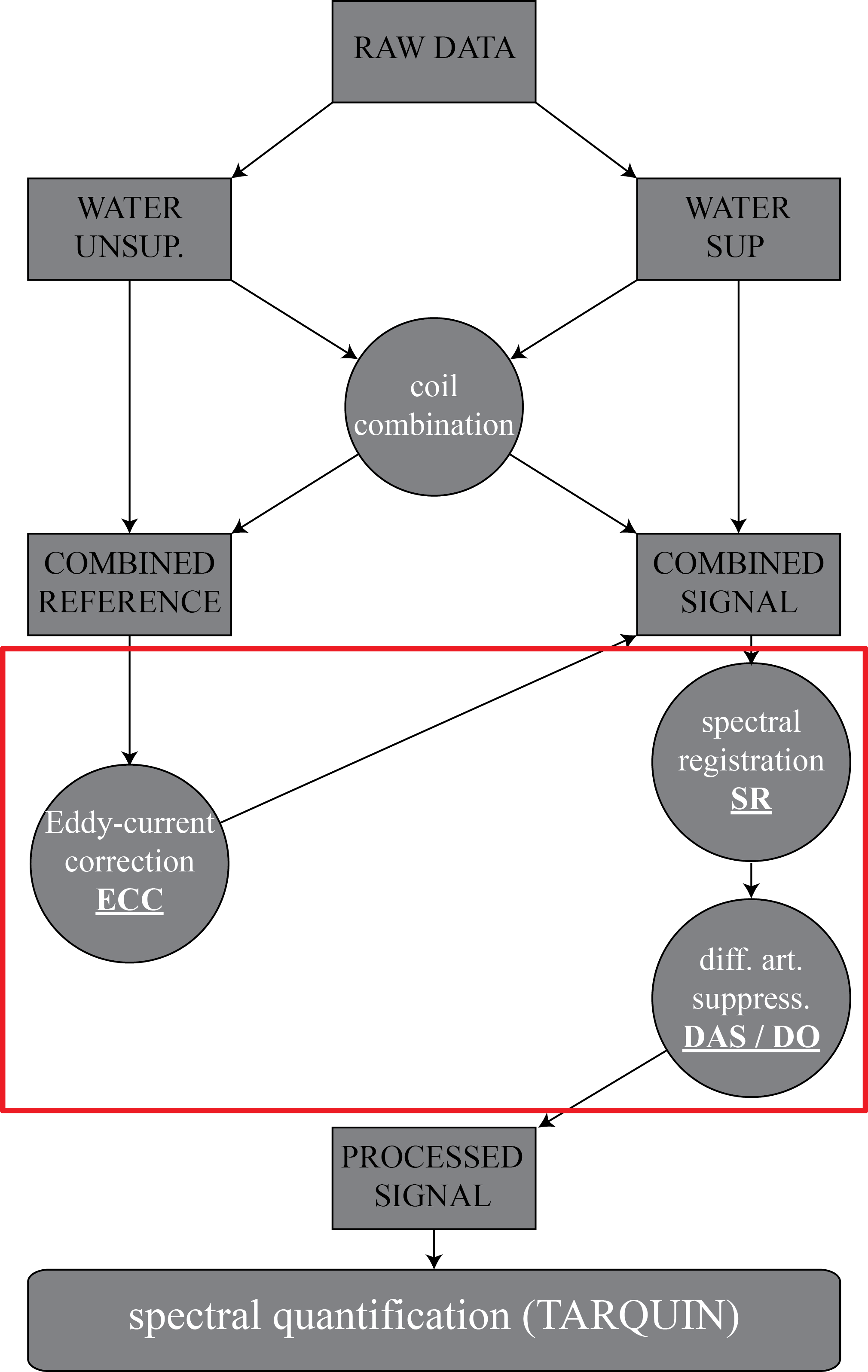

Supplement: Supplementary file 1 — Supplementary figure 1: Flowchart depicting data preprocessing. Raw data are read in and split into water-suppressed and water-unsuppressed signal. Both signals are used for coil combination. In the red box, optional preprocessing steps are shown. This includes eddy current compensation, spectral registration and difference artifact suppression. The resulting processed data are exported in JMRUI file format and imported by TARQUIN for spectral registration [file 10334_2020_879_MOESM1_ESM.png]

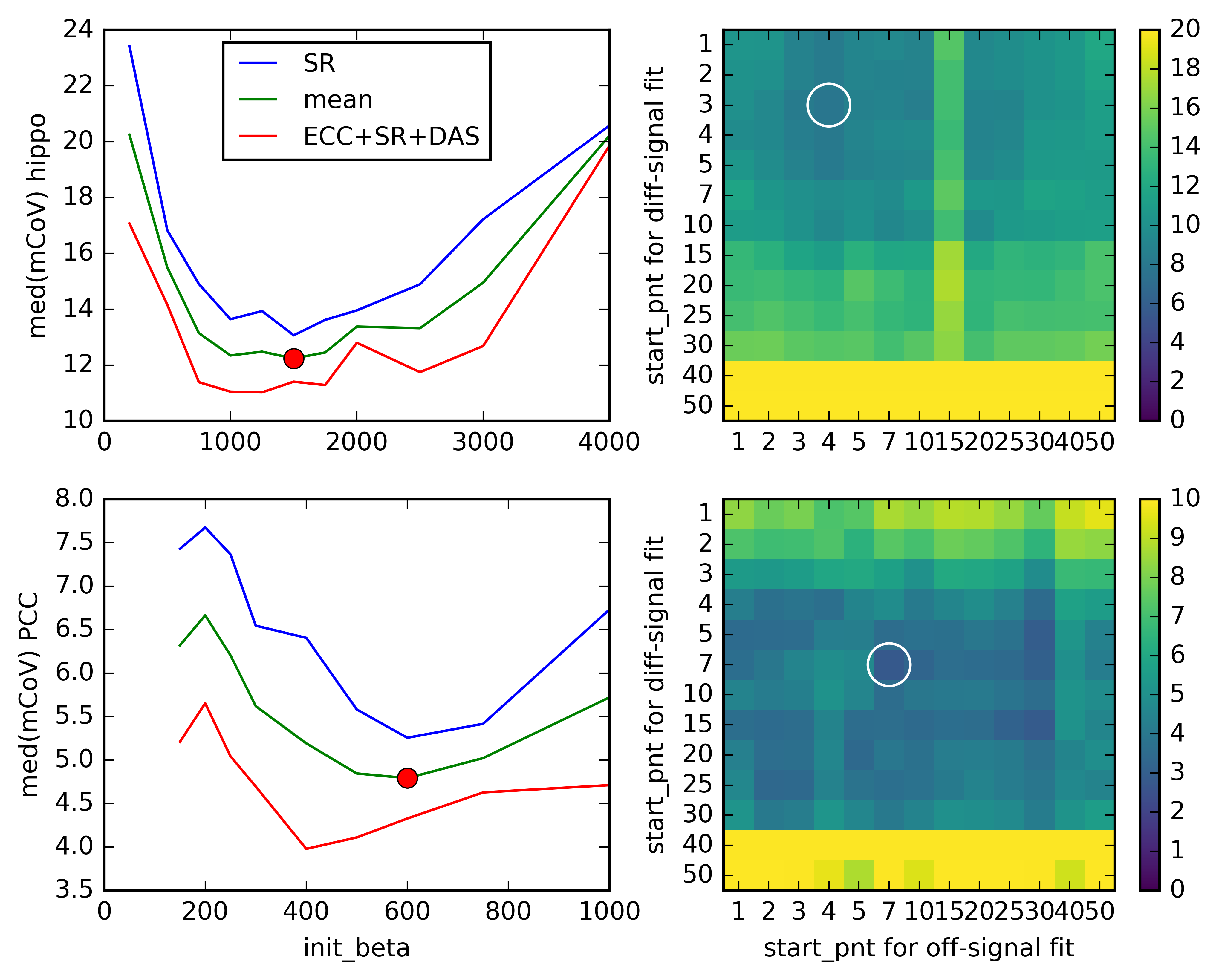

Supplement: Supplementary file 2 — Supplementary figure 2: Results of TARQUIN parameter optimization. Left: median intra-session mCoV over all pairs of $n_s$ns as a function of $\beta _s$βs. It was calculated for the processing routines SR (standard processing) and ECC + SR + DAS (advanced processing). The minimum of the average of this two values were reached at $\beta _s=1500$βs=1500 (hippocampus) and $\beta _s=600$βs=600 (PCC). Right: median intra-session mCoV over all processing approaches for various combinations of $n_s$ns, using optimized $\beta _s$βs. The w [file 10334_2020_879_MOESM2_ESM.png]
